# The preparation of prochloraz pH-responsive nanocapsules by the Pickering emulsion polymerization method and the study of their performance

**DOI:** 10.1039/c9ra09920d

**Published:** 2020-01-28

**Authors:** Fei Xue, Ziwei Zhu, Zheng Wei, Xinya Peng, Yalan Wang, Tian Li, Guanhua Ma, Yan Wu, Lin He, Kun Qian

**Affiliations:** College of Plant Protection, Southwest University No. 2 Tiansheng Road Chongqing 400715 China qiankun1982@163.com helinok@vip.tom.com; National Center for Nanoscience and Technology Beijing 100190 China wuy@nanoctr.cn; Academy of Agricultural Sciences, Southwest University Chongqing 400715 China

## Abstract

In this work, prochloraz pH-responsive nanocapsules were developed by the Pickering emulsion polymerization method with isophorone diisocyanate (IPDI) as the reaction monomer and nano Fe_3_O_4_ particle-branched polyethyleneimine (PEI) as the reaction monomer and surfactant. The physical and chemical properties and sustained release properties were determined by a transmission electron microscope (TEM), field emission transmission electron microscope (FETEM), atomic force microscope (AFM), laser particle size analyzer, Fourier transform infrared spectrometer, and contact angle tester. The results indicated that the prochloraz nanocapsules were spherical, the average particle size was about 100 nm, and the encapsulation efficiency and loading rates were 86% and 30%, respectively. The nanocapsules tended to expand in acidic solutions, and this promoted the release of prochloraz more quickly, which could be verified by the biological test of anthrax. At the same time, the prochloraz nanocapsules can protect the pesticide from sunlight. Therefore, this work provides a promising approach to improve the utilization efficiency and prolong the duration of pesticides, which might have a huge potential application prospect.

## Abstract 2

In this work, prochloraz pH-responsive nanocapsules were developed by the Pickering emulsion polymerization method with isophorone diisocyanate (IPDI) and nano Fe_3_O_4_ particle-branched polyethyleneimine (PEI).

## Graphical abstract

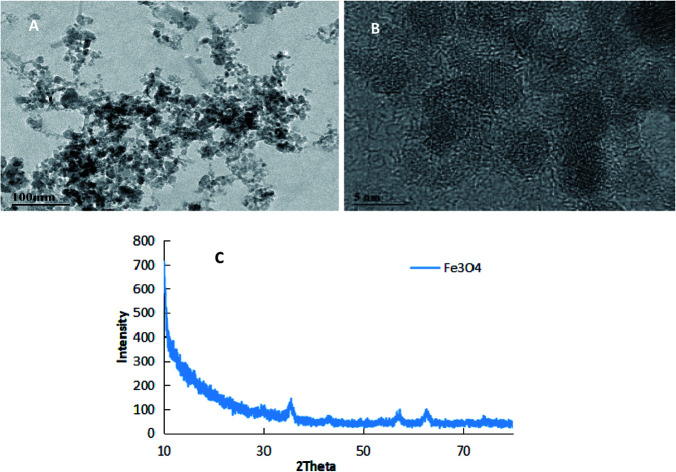

## 1. Introduction

Prochloraz [*N*-propyl-*N*-[2-(2,4,6-trichlorophenoxy)ethyl]imidazole-1-carboxamide, PCZ] belongs to the group of imidazole fungicides that inhibit ergosterol biosynthesis. It is widely used to control eyespot disease and powdery mildew on cereals, and it is also effective against a broad spectrum of fungal diseases affecting fruits and vegetables.^1–3^ It has been reported that the half-life of prochloraz after photolysis in an aqueous solution is 10 days. The dissipation half-lives in soil range between 5 and 37 days under field conditions. It is therefore necessary to develop a novel formulation to increase the stability of prochloraz in the environment.

Pesticides are considered as the most effective way to control weeds, pests, and diseases in modern agriculture for the promotion of grain yields. However, traditional pesticides tend to enter the environment easily through runoff, volatilization, and leaching, leading to serious environmental issues and even hazards to human beings.^4^ Consequently, it is urgent to develop new approaches to reduce the loss and improve the utilization efficiency of pesticides. A promising method to resolve this problem involves the construction of controlled-release pesticide systems, which will fulfill the demand of prolonging the duration and enhancing the utilization efficiency of the pesticides.^5–9^

Recently, controlled-release pesticides are attracting more and more attention all over the world.^10,11^ Accordingly, various kinds of nanomaterials have been used as carriers in these controlled-release pesticide systems such as polymers,^12,13^ inorganic nanomaterials,^14^ and nanocomposites.^15^ There are some chemical preparation methods for synthesizing nanocapsules, such as *in situ* polymerization, suspension polymerization, interfacial polymerization, and emulsion polymerization. Pickering emulsion polymerization possesses many advantages including the reduction of foaming, lower toxicity and lower cost, and it has been extensively studied. Solid particles as surfactants and fillers provide a direction to prepare stable Pickering emulsions, such as nano-SiO_2_ particles and nanocomposite latex particles. Magnetic nanoparticles have been widely studied because of their fascinating magnetic separation properties and wide range of potential applications in pigments, medicine, biomedical and bioengineering fields, *etc.*^16–19^

At the same time, a pesticide needs to be released quickly in environmental conditions (such as pH and temperature) when a plant disease occurs and in the presence of insect pests. Thus, environmental-responsive pesticide capsules should be designed to meet the requirements for controlling pests. Many diseased plants can secrete oxalic acid and other compounds during disease development, resulting in a slightly acidic environment. At this point, we designed a capsule that can be rapidly released under acidic conditions. At the same time, this capsule can maintain the drug's stability and its effectiveness to achieve its quick action and timely prevention and control of diseases.

In our study, the anthracnose pathogen, a widely present fungus, was used as the target, and 5% prochloraz pH-responsive nanocapsules were prepared by the Pickering emulsion polymerization method with isophorone diisocyanate (IPDI) and polyethyleneimine (PEI)-modified nano-Fe_3_O_4_ as the reaction monomer and emulsifier, respectively. Meanwhile, the release kinetics, light stability, and efficacy of the prochloraz nanocapsules against the anthracnose pathogen are studied further.

## 2. Material and method

### 2.1. Materials

The model pesticide prochloraz (purity 98%) was supplied by Hubei Jusheng Technology Co., Ltd. (Hubei, China). IPDI (purity 99%) was purchased from Jining Hongming Chemical Reagent Co., Ltd (Shandong, China). PEI (purity 99%) was purchased from Shanghai Tengzhun Biotechnology Co., Ltd. Tristyrylphenol ethoxylate (600#) was purchased from Zhongyue Chemical Technology Co., Ltd (Nanjing, China). Xylene, FeCl_2_·4H_2_O, FeCl_3_·6H_2_O and NaOH were analytical chemicals purchased from Chengdu Kelong Chemical Reagent Factory (Sichuan, China). Acetonitrile and methanol were HPLC grade and purchased from J. T. Baker (USA).

### 2.2. The preparation and characterization of nano-Fe_3_O_4_

In this study, magnetic nano-Fe_3_O_4_ was prepared by chemical co-precipitation.^20^ First, 4.4 g FeCl_2_·4H_2_O was dissolved in 10 mL deionized water and filtered by a membrane; 5.2 g FeCl_3_·6H_2_O was dissolved in 100 mL deionized water. Then, the FeCl_3_ solution was added into the flask, stirred and heated to 70 °C; 7 mL FeCl_2_ solution was added into the flask, and 12 mL concentrated ammonia water with 25% mass fraction was added rapidly under intense stirring. After 1 hour, a black sol-like liquid was obtained, separated by a magnet, and washed several times with methanol and water, and the samples were freeze-dried. Then, 1 g PEI was dissolved in 100 mL deionized water, and 1 g nano-Fe_3_O_4_ was added to the solution and stirred to form a brown sol-like liquid. The samples were separated by magnets, washed several times with deionized water, and freeze-dried to obtain PEI-modified nano-Fe_3_O_4_. The physical and chemical properties of PEI-modified nano-Fe_3_O_4_ were characterized by TEM and Fourier transform infrared spectroscopy.

### 2.3. The preparation and characterization of prochloraz nanocapsules

PEI-nano-Fe_3_O_4_ (0.1 g) was added to 14 g deionized water containing 100 μL acetic acid and stirred to form an aqueous phase. Then, 1 g of prochloraz, 0.4 g of 600# and 2 g of IPDI were dissolved in 1 g of xylene to form the oil phase. After that, the oil phase was added into the water phase dropwise under stirring. Prochloraz nanocapsules were obtained after stirring for 6 h for full reaction of IPDI and PEI. The physical and chemical properties and sustained release properties of prochloraz nanocapsules were characterized by TEM, FETEM, laser particle size analyzer, Fourier transform infrared spectroscopy, HPLC and contact angle tester.

### 2.4. Determination of encapsulation efficiency and loading rate of prochloraz nanocapsules

The prochloraz nanocapsules were washed and centrifuged, and the precipitated capsules were dried to form powder capsules. Dry prochloraz nanocapsules were weighed and placed in the test tube. An appropriate amount of methanol was added and prochloraz was completely dissolved in methanol by ultrasonic dispersion. Then, the samples were centrifuged at a high speed and the supernatant was taken to prepare the samples after filtering. The concentration of prochloraz was determined by HPLC. The chromatographic conditions are as follows: mobile phase: methanol and water, volume ratio: 80 : 20, flow rate: 1 mL min^−1^, temperature: 40 °C, detection wavelength: 220 nm, injection volume: 10 μL. The encapsulation efficiency and drug loading rate of nanocapsules were calculated by the following formulas.Encapsulation efficiency = the mass of prochloraz in nanocapsules/total dosage of prochlorazLoading rate = the mass of prochloraz in nanocapsules/the mass of nanocapsules

### 2.5. Light stability investigation

Prochloraz nanocapsules (5 mL) and EC were added to a Petri dish and placed under an incandescent lamp. Methanol was added to the Petri dish after 3, 5 and 7 days. The active ingredients were extracted repeatedly by ultrasound and detected by liquid chromatography. Then, the degradation ratio (DR) of prochloraz in the nanocapsules and EC was calculated as follows:DR = (*A*_0_ − *A*_*t*_)/*A*_0_ × 100%Here, *A*_*t*_ is the residue amount of prochloraz at time of *t*, and *A*_0_ is the initial amount of prochloraz.

### 2.6. Indoor bioassay

The tested strain is anthracnose pathogen, which was cultured in the College of Plant Protection, Southwest University. Citrus anthracnose fungus was inoculated on sterilized potato dextrose agar (PDA)^21^ and cultured for 144 hours at 25 °C so as to increase the strain reserve. The nanocapsules were diluted at different concentrations and mixed into the sterilized melt PDA medium. The PDA medium with different concentrations of prochloraz was prepared by pouring into a 9 cm diameter dish and cooling. Sterilized water was added to the PDA medium as the CK. Each sample was repeated three times in the experiment. After PDA was solidified, the bacterial samples were punched with a 6 mm diameter perforator and then inserted into the PDA medium. After 144 hours of culture, the colony diameters were determined by the cross-over method. The inhibition rate is calculated by using the formula and the toxicity curve and the median effect concentration (EC_50_) was obtained. Certain amounts of the diluted prochloraz nanocapsule solution and emulsifiable concentrate solution were added to the melt PDA medium with pH 5.5, 7 and 8.5. The concentration of prochloraz in PDA was EC_50_. Each sample was repeated three times in the experiment. The anthracnose pathogen was cultured by the same method, and the inhibition efficiency was calculated after 7 days.
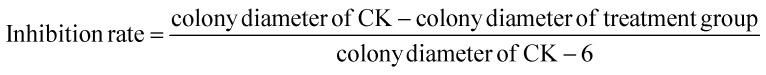


## 3. Result and discussion

### 3.1. The preparation and characterization of nano-Fe_3_O_4_ and prochloraz nanocapsules

**Fig. 1:**
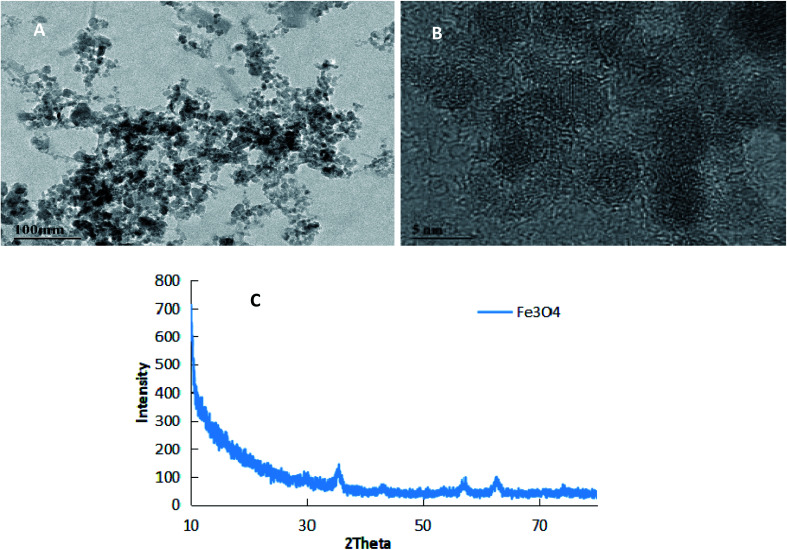
The TEM images (A and B) and XRD image (C) of Fe_3_O_4_ nanoparticles.

Fig. 1 shows the TEM microscopy (A), FETEM microscopy (B) and XRD (C) results of PEI-nano-Fe_3_O_4_. From the figure, we can see that nano-Fe_3_O_4_ is evenly distributed and the average particle sizes are in the range of 5–10 nm. Comparing Fig. 1A and B, we can find that PEI-nano-Fe_3_O_4_ has better dispersibility. This also indicated that PEI successfully modified nano-Fe_3_O_4_.

### 3.2. The morphology, particle size distribution and magnetic separation of prochloraz nanocapsules

**Fig. 2:**
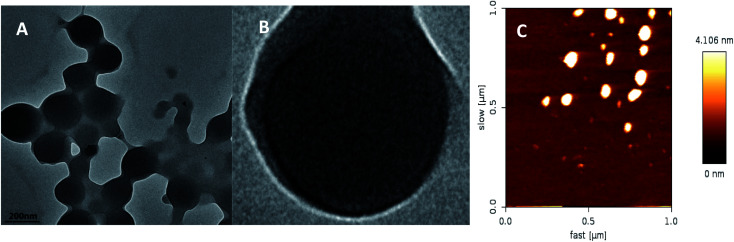
The TEM image (A), FETEM image (B) and AFM image (C) of prochloraz nanocapsules.

**Fig. 3:**
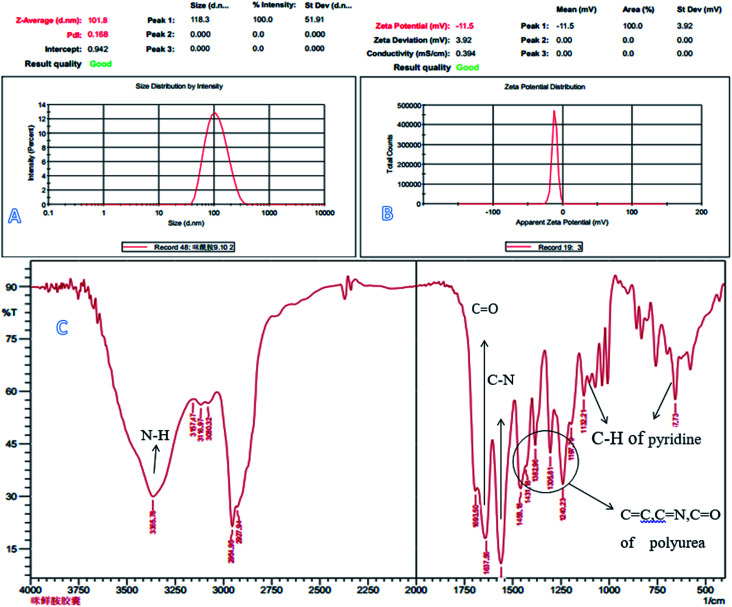
The laser particle size analyzer image (A), zeta potential (B) and IR patterns (C) of prochloraz nanocapsules.

Fig. 2 shows the TEM images (A), FETEM images (B) and AFM images (C) of prochloraz capsules. It can be seen from the figure that the prochloraz nanocapsules are uniformly distributed and have a similar shape to that of spheres and a granular bulge on the surface because of the existence of PEI-nano-Fe_3_O_4_ on the surface of the capsules. The particle size is about 80–130 nm, which can also be proven in Fig. 3. After 15 days, there was no obvious change in the particle size, which indicated that the prochloraz nanocapsules were stable. The encapsulation efficiency and loading rate were 86% and 30% by the formula, respectively. In order to explore the recovery potential, nanocapsules were adsorbed and separated by magnets and then, the content of active ingredients in the remaining liquid was determined. We found that the content of the active components in the remaining liquid was about 48.7% of the total amount, and the adsorption recovery was more than 50%.

Fig. 3 shows the laser particle size analyzer images (A), zeta potential (B) and infrared spectra (C) of the prochloraz nanocapsules. It can be seen from the figure that the average particle size of cyhalothrin nanocapsules is 100 nm, and the zeta potential of the capsules is concentrated in the negative region. This shows that there are same charges on the surface of the capsules and they repel each other, which is one of the reasons for the stability of the suspensions. In Fig. 3C, the peak at 3365 cm^−1^ stands for N–H of the polyurea stretching vibration. The absorption peaks at 1240–1458 cm^−1^ are due to the C=O, C=N, and C=C stretching vibrations and N–H and C–H flexural vibrations. These peaks prove that IPDI and PEI-nano-Fe_3_O_4_ react to form polyurea. For prochloraz, the main characteristic infrared absorption peaks were attributed to the C=O stretching (1637 cm^−1^), C–N stretching (1567 cm^−1^) and C–H of pyridine flexural vibration (1132, 657 cm^−1^). All of the characteristic absorption peaks can be found for the prochloraz nanocapsules, which indicate that prochloraz is successfully encapsulated in the nanocapsules.

### 3.3. Contact angle and adhesion work of prochloraz nanocapsules

**Fig. 4:**
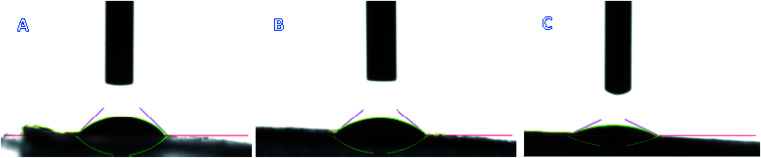
The contact angles of three types formulations: EC (A), EW (B) and nanocapsules (C).

**Fig. 5:**
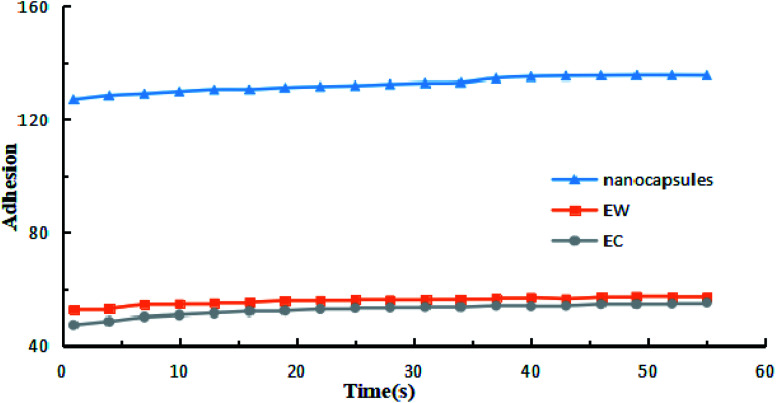
The relationship between work of adhesion and time when three kinds of formulations were diluted to 200 mg L^−1^.

The contact angles of the prochloraz emulsion concentrate (EC) (A), emulsion in water (EW) (B) and nanocapsules (C) on cowpea leaves are shown in Fig. 4. The prochloraz nanocapsules, EC and EW were diluted to 200 mg L^−1^ and their contact angles were measured. We found that the contact angles of EC, EW and nanocapsules were 45.52°, 40.58°, and 25.28°, respectively. The contact angles of the nanocapsules were significantly smaller than those of EC and EW, indicating that the nanocapsules had better wettability. At the same time, we analyzed the relationship between the adhesion work and time under different dosage forms, as shown in Fig. 5. It can be seen that under the same content of active ingredients, the adhesion work increases gradually with the increase in time, and the work of adhesion of prochloraz nanocapsules is significantly higher than that of the other two groups. The results showed that compared with EC and EW, the prochloraz nanocapsules did not easily fall off from the leaves, and had a stronger anti-rainwater scouring effect, which could improve the effective utilization rate of prochloraz.

### 3.4. Release kinetics and stability of prochloraz nanocapsules

#### 3.4.1. The effects of different pH on the release behavior of prochloraz nanocapsules

**Fig. 6:**
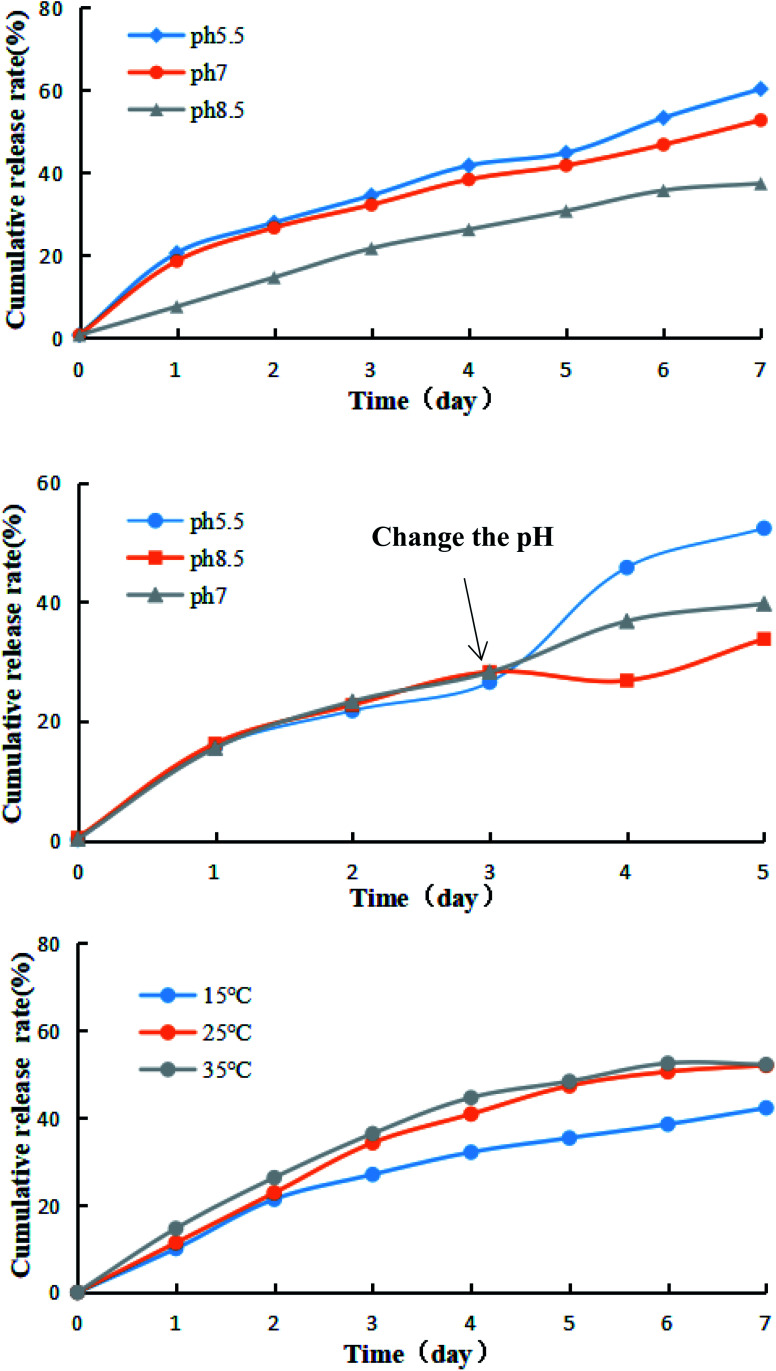
The release curves of prochloraz nanocapsules under different conditions.

**Scheme 1:**
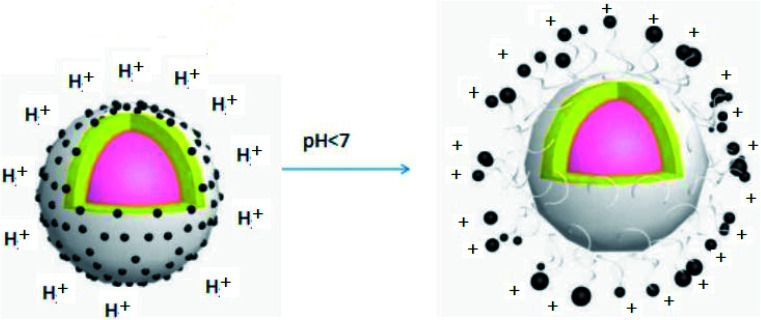
The release mechanism of prochloraz nanocapsules.

**Fig. 7:**
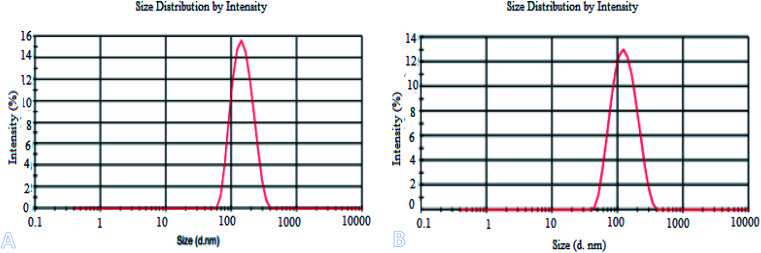
The laser particle size analyzer images under acidic (A) and alkaline (B) conditions.

Certain amounts of the prochloraz nanocapsules were packed in dialysis bags (MW: 3500). The release behavior and stability of the prochloraz nanocapsules were studied by changing the pH under the condition that the stirring speed was 100 rpm and the temperature was kept constant (25 °C). Fig. 6 shows the effects of different pH values (5.5, 7.0 and 8.5) on the release behavior and stability of the prochloraz nanocapsules. The cumulative release rates of the prochloraz nanocapsules were from 21.6% to 34.5% on the 3rd day, 30.7% to 44.8% on the 5th day and 37.4% to 60.2% on the 7th day. The release rate of the prochloraz nanocapsules was faster under acidic conditions. To verify this, we experimented by changing the pH of the medium during the release process. The results are shown in Fig. 6. The release rates of the three groups of prochloraz nanocapsules were almost the same in the first 3 days without changing the neutral medium. However, within 24 hours of changing the pH, the release of prochloraz in the acidic medium was 2.2 times as much as that in the neutral medium. The content of prochloraz in the alkaline medium decreased first and then increased, which was related to the decomposition of prochloraz in the alkaline medium. Therefore, prochloraz nanocapsules can be released rapidly in acidic conditions. This may be due to the presence of a large number of –NH– species in PEI-nano-Fe_3_O_4_, which bind with H^+^ to produce a repulsive force and enlarge the capsule gap. At the same time, due to the negative zeta potential of the capsule as a whole, the increase in H^+^ will also destroy the potential balance and make the capsule unstable. The release mechanism of the prochloraz nanocapsules is shown in Scheme 1. In order to verify the effect of pH on the capsule size, we determined the capsule size under different pH values. The results are shown in Fig. 7A (5.5) and Fig. 7B (8.5). We found that under acidic and alkaline conditions, the particle sizes of the nanocapsules were 140 nm and 115 nm, respectively. Compared with the result shown in Fig. 3A, the particle sizes of the nanocapsules increased by 40% and 15%.

#### 3.4.2. The effects of different temperatures on the release behavior of prochloraz nanocapsules

Certain amounts of prochloraz nanocapsules were packed in dialysis bags (MW: 3500). The release behavior and stability of the prochloraz nanocapsules were studied by changing the temperature under the condition that the stirring speed was 100 rpm and the pH was kept constant (pH = 7). Fig. 6 shows the effects of different temperatures (15 °C, 25 °C and 35 °C) on the release behavior and stability of prochloraz nanocapsules. The cumulative release rates of prochloraz nanocapsules were from 27.0% to 36.4% on the 3rd day, 35.4% to 48.4% on the 5th day and 42.3% to 52.2% on the 7th day. The results indicated that the thermal motion of molecules was more violent, which resulted in an easier and quicker release of the prochloraz nanocapsules at a higher temperature.

#### 3.4.3. Release kinetics of prochloraz nanocapsules

The origin software was used to fit the simulation equation of the cumulative release rate under different pH values. The zero order equation, first order equation, Higuchi equation and Peppas equation were chosen as the fitting models.^22–24^ It can be seen from Table 1 that the degree of the fitting of the release kinetics of the prochloraz nanocapsules with the Peppas equation is the highest, which indicates that the Peppas equation can more accurately describe the release of the prochloraz nanocapsules.

**Table 1:** Fitting models for prochloraz nanocapsules at different temperatures and pH

Condition		Equation			
		Fitting degree			
		Zero order equation *Q* = *Q*_0_ + *kt*	First order equation *Q* = *a*(1 − e^−*kt*^)	Higuchi equation *Q* = *kt*^1/2^	Peppas equation *Q* = *kt*^*n*^
pH	pH = 5.5	0.8783	0.9821	0.7346	0.9870
	pH = 7	0.8353	0.9946	0.6117	0.9973
	pH = 8.5	0.9590	0.9876	0.9308	0.9910
Temperature	15 °C	0.8853	0.9588	0.7668	0.9647
	25 °C	0.9092	0.9602	0.8311	0.9707
	35 °C	0.8488	0.9375	0.6837	0.9689

Further results for fitting with the Peppas equation are shown in Table 2. Here, *n* is an index, which reflects the release mechanism: Fickian diffusion (*n* < 0.43), non-Fickian or anomalous diffusion (0.43 < *n* < 0.85), and case II transport (*n* > 0.85).^25^ The results showed that the diffusion index *n* was between 0.43 and 0.85 at different pH values; thus, the release of nanocapsules belonged to non-Fickian or anomalous diffusion.

**Table 2:** Fitting Peppas models of prochloraz nanocapsules at different temperatures and pH

Condition	pH/temperature	*k*	*n*	Correlation coefficient (*R*^2^)
pH	5.5	19.6858	0.5440	0.9870
	7	18.4599	0.5224	0.9973
	8.5	8.0494	0.8298	0.9910
Temperature	15 °C	11.7470	0.7088	0.9647
	25 °C	12.6278	0.7960	0.9707
	35 °C	15.9698	0.6744	0.9689

### 3.5. Stability investigation under incandescent light

**Fig. 8:**
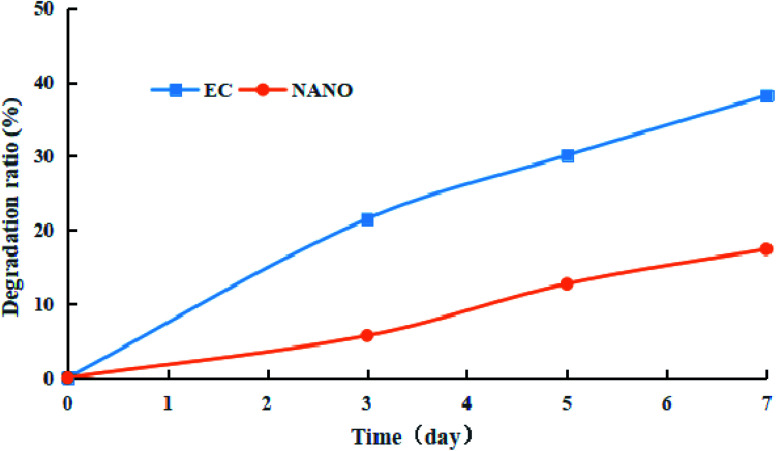
Degradation ratios of EC and nanocapsules under incandescent light irradiation.

Owing to the low light stability, prochloraz tends to degrade under incandescent light irradiation, resulting in low utilization efficiency. In order to obtain light stability, the prochloraz degradation ratios of nanocapsules and EC were investigated under incandescent light irradiation. As shown in Fig. 8, the prochloraz degradation ratio of EC increases with time and reaches up to 38.2% after 7 days, while the prochloraz degradation ratio of the nanocapsules on the 7th day is just 17.4%. The degradation rate is about 21% lower than that of EC. Therefore, the prochloraz nanocapsules can effectively alleviate the photolysis of prochloraz and improve the stability.

### 3.6. Indoor bioassay

**Fig. 9:**
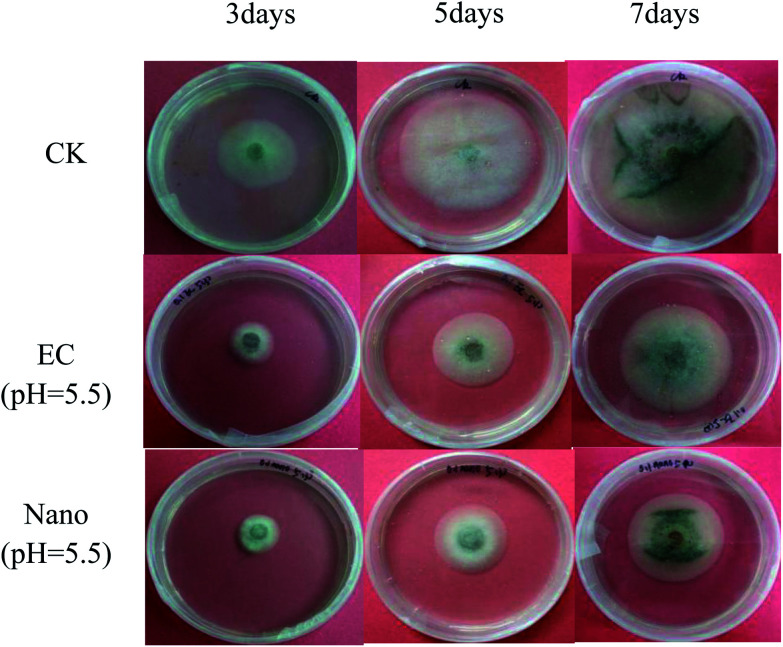
Laboratory biometric pictures of prochloraz EC and nanocapsules.

In the indoor bioassay experiment, we first set up a series of concentration gradients to fit the toxicity curve of prochloraz to the anthracnose pathogen and calculated EC_50_ to be about 0.1 mg L^−1^. Then, we carried out the inhibition test of the nanocapsules and EC for the fungus. The results are shown in Fig. 9 and Table 3.

**Table 3:** The inhibition rate of prochloraz nanocapsules for *Colletotrichum gloeosporioides* Pen.^a^

Dosage form	pH	Inhibition rate (%)		
		3 d	5 d	7 d
5% prochloraz nanocapsules	5.5	43.9 ± 1.2c	52.4 ± 1.5a	48.8 ± 0.7a
	7	45.8 ± 2.0c	51.5 ± 1.7a	47.7 ± 0.3a
	8.5	46.1 ± 2.6c	51.1 ± 1.2a	45.3 ± 1.1b
20% prochloraz EC	5.5	59.8 ± 1.5a	49.8 ± 3.6a	38.5 ± 1.1d
	7	56.9 ± 5.2ab	51.6 ± 2.1a	41.1 ± 1.3c
	8.5	55.5 ± 3.5b	50.4 ± 1.5a	42.1 ± 1.1c

a Values marked with the same letters are not significantly different.

From the chart, we find that the inhibition rate of EC shows a downward trend and the inhibition rate of the nanocapsules at the 7th day is significantly higher than that of EC, which may be ascribed to the photolysis of prochloraz in EC during the culture process; also, the prochloraz nanocapsules can effectively alleviate the photolysis of prochloraz. The inhibition rates at different pH values (pH 5.5, 7 and 8.5) were 48.8%, 47.7%, and 45.3% on the 7th day, respectively. The results showed that the inhibition rate of nanocapsules under acidic conditions was higher than that under alkaline conditions on the 7th day. This may be because the prochloraz nanocapsules are released faster under acidic conditions.

## 4. Conclusion

In this work, prochloraz pH-responsive nanocapsules were developed by the Pickering emulsion polymerization method. The release rate of the prochloraz nanocapsules is faster under acidic conditions and the release of the nanocapsules belongs to non-Fickian or anomalous diffusion. The nanocapsules have excellent magnetic separation performance (more than 50%). In addition, they can prevent the photolysis of prochloraz. The results of an indoor bioassay also indicated that the inhibition rate of 5% prochloraz nanocapsules was significantly higher than that of EC on the 7th day.

## Conflicts of interest

There are no conflicts to declare.

## Supplementary Material
